# Quality of life and tumor control after short split-course chemoradiation for anal canal carcinoma

**DOI:** 10.1186/1748-717X-5-41

**Published:** 2010-05-23

**Authors:** Sawyna Provencher, Christoph Oehler, Sophie Lavertu, Marjory Jolicoeur, Bernard Fortin, David Donath

**Affiliations:** 1 Department of Radiation Oncology, Centre Hospitalier Universitaire de Montréal- Notre-Dame Hospital, Canada; 2 Department of Radiation Oncology, Centre Hospitalier Universitaire de Sherbrooke, Canada; 3 Department of Radiation Oncology, University Hospital Zurich, Switzerland

## Abstract

**Purpose:**

To evaluate quality of life (QOL) and outcome of patients with anal carcinoma treated with short split-course chemoradiation (CRT).

**Methods:**

From 1991 to 2005, 58 patients with anal cancer were curatively treated with CRT. External beam radiotherapy (52 Gy/26 fractions) with elective groin irradiation (24 Gy) was applied in 2 series divided by a median gap of 12 days. Chemotherapy including fluorouracil and Mitomycin-C was delivered in two sequences. Long-term QOL was assessed using the site-specific EORTC QLQ-CR29 and the global QLQ-C30 questionnaires.

**Results:**

Five-year local control, colostomy-free survival, and overall survival were 78%, 94% and 80%, respectively. The global QOL score according to the QLQ-C30 was good with 70 out of 100. The QLQ-CR29 questionnaire revealed that 77% of patients were mostly satisfied with their body image. Significant anal pain or fecal incontinence was infrequently reported. Skin toxicity grade 3 or 4 was present in 76% of patients and erectile dysfunction was reported in 100% of male patients.

**Conclusions:**

Short split-course CRT for anal carcinoma seems to be associated with good local control, survival and long-term global QOL. However, it is also associated with severe acute skin toxicity and sexual dysfunction. Implementation of modern techniques such as intensity-modulated radiation therapy (IMRT) might be considered to reduce toxicity.

## Introduction

Sphincter-sparing chemoradiation (CRT) has evolved as the standard of care for most patients with squamous cell carcinoma of the anal canal. Combined CRT was first introduced by Nigro et al. in the mid-1970s, and has resulted in improved local and regional control, colostomy free survival, and disease-free survival since then [1-4]. Currently, local tumor control and disease-free survival have approximated 72% and 73%, respectively, in randomized trials [4]. Mortality to incidence ratio was 14% (660 estimated deaths in the United States in 2006) implying that the majority of patients with anal cancer have a good prognosis [5]. Given these results, coupled with the preservation of the rectum itself, maintaining satisfactory ano-rectal function and controlling toxicity have become important parameters in the evaluation of CRT.

Sphincter-conserving CRT is associated with considerable acute and chronic complications. Split-course radiotherapy with a planned gap was initially implemented to select poor responders for surgery and good responders for boosting either with external beam radiotherapy (EBRT) or preferably brachytherapy. Split duration was 6 weeks, but was reduced in the recent years. Since there were toxicity concerns the feasibility of reducing the gap between sequences to 2 weeks was tested by the EORTC phase II study 22953 [6]. Though acute toxicities have been reported to be moderate and long-term toxicities to be acceptable after 3 years, long-term QOL has not yet been evaluated after this regimen.

This study was conducted to evaluate QOL using the newly implemented site-specific questionnaire QLQ-CR29 of patients with anal canal cancer treated with short split-course radiotherapy and concurrent chemotherapy with fluorouracil (5-FU) and Mitomycin C (MMC). Secondary endpoints were acute toxicity, local and regional tumor control, colostomy-free survival and overall survival.

## Methods

### Patient characteristics

From 1991 to 2005, 58 consecutive patients with non-metastatic cancer of the anal canal were treated curatively with a short split-course of CRT at the Centre Hospitalier de l'Université de Montréal (CHUM), Notre-Dame Hospital. The histopathological diagnosis was established according to the World Health Organization (WHO) criteria [7]. Adenocarcinoma of the anal canal was excluded because of its different behavior, management and prognosis [8,9]. All patients had a complete work-up including chest x-ray, CT-scan of the abdomen and pelvis, blood analyses and rectoscopy with a tissue biopsy.

### Treatment

All patients received curative CRT. Standard 3-dimensional conformal whole pelvis external beam radiotherapy (EBRT), using photons of 6 MV to 18 MV, was delivered in two series. The first series of 24 Gy in 12 fractions was delivered via anterior and posterior parallel opposed fields and encompassed macroscopic and microscopic disease. Larger rectangular AP/PA fields with distal margin set to include the anal canal were used to include the primary anal tumour, involved nodes and nodal areas at risk including perirectal, external and internal iliac and inguinal lymph nodes. After a planned break of a median of 12 days, the second series of EBRT (28 Gy/14 fractions) was applied to the macroscopic disease up to a total dose of 52 Gy. Chemotherapy consisted of 5-FU delivered at a dose of 1000 mg/m2/day over 120 hours of continuous intravenous infusion on days 1 to 5 and days 29 to 33. At the same time, MMC was given at 10 mg/m2/day on day 1 and 29. Both were delivered in the first week of each radiation therapy series.

Acute toxicity was retrospectively evaluated according to the RTOG acute toxicity scale using the patient's chart.

### QOL assessment

The long-term QOL was assessed by two standardized EORTC questionnaires. The first one, QLQ-C30 version 3.0, is a validated questionnaire assessing cancer-specific QOL [10]. The second one, QLQ-CR29, assesses site specific (ano-rectal) QOL [11]. The EORTC QLQ-CR29 questionnaire is based on EORTC QLQ-CR38 questionnaire and has been recently updated and modified based on evidence from the literature, expert opinion and interviews with patients [11]. This study used the first version of the QLQ-CR29 questionnaire. This module is a self-rating questionnaire that comprises 29 questions. Two questions are specific for patients with stoma and 2 more questions are directed only to females or males. The principal items of this questionnaire include urinary symptoms, pain, fecal incontinence, gastro-intestinal function, stoma, male and female sex and body image. For all the questions, a scale from 1 to 4 was used (1: not at all, 2: a little, 3: quite a bit, 4: very much).

### Statistics

All survival analyses were calculated according to the Kaplan-Meier method. Overall survival was calculated from time to diagnosis to death from any cause. Disease-free survival was measured from date of diagnosis to recurrence or death from all causes, or censored at last to follow-up. Multivariate analysis was performed using the Cox regression model. The variables tested were gender, HIV status, T3-T4 vs T1-T2, N0 vs N-positive, 0-1 cycle of 5-FU vs ≥ 2 cycles of 5-FU and 0-1 cycle of MMC vs ≥ 2 cycles of MMC. The SAS program was used for scoring the QLQ-C30 according to EORTC QLQ-C30 scoring manual [12]. All scores of the QLQ-C30 were linearly transformed such that all scales range from 0 to 100. For the six functional items, the higher score represents a higher level of functioning and for the symptoms/single items; a higher score means a higher level of symptomatology/problems. For the QLQ-CR29, each question was analyzed independently using the scale from 0 to 4 mentioned above.

## Results

Among the 58 patients reported, 32 patients were female and 26 patients were male with a median age of 53 years (range 36-84). Fifty-seven patients had squamous-cell carcinoma and 1 patient had undifferentiated carcinoma. Patient characteristics are summarized in Table 1. Six patients (10%) were HIV-positive and were receiving highly active antiretroviral treatment. The T-Stage distribution, according to the 2001 American Joint Committee on Cancer/TNM classification, was T1 (9; 16%), T2 (22; 38%), T3 (13; 22%), T4 (13; 22%) and Tx (1; 2%) [13]. The distribution according to the N-Stage was N0 (42; 72%), N1 (5; 9%), N2 (5; 9%) and N3 (6; 10%). Sixteen percent (9 of 58 patients) had inguinal nodal involvement.

**Table 1:** Patient caracteristics

	Number of patients (%)	N-Stage			
		N0	N1	N2	N3
**Gender**					
F	32 (55)				
M	26 (45)				
Median age (years)	53 (36-84)				
HIV+	6 (10)				
Histology					
Squamous cell	57 (98)				
undifferentiated	1 (2)				
T-Stage					
T1	9 (16)	9	0	0	0
T2	22 (38)	17	1	2	2
T3	13 (22)	7	2	2	2
T4	13 (22)	9	2	1	1
Tx	1 (2)	0	0	0	1

Patient characteristics (n = 58). F = female, M = male.

All but one (because of morbid obesity) patient with negative inguinal lymph nodes received prophylactic EBRT to the bilateral groins at a median dose of 24 Gy (range 20-30 Gy). The median duration of the planned break was 12 days (11-13 days (25-75 quartiles)). For the second sequence of radiation therapy, most of the patients were treated with a 3-field technique (41%), 4-field technique (40%) or an AP/PA technique (19%). The total radiation dose delivered to the macroscopic disease was 52 Gy (range 42-78 Gy). The median duration of the CRT was 48 days (range 42-78). Two patients with a T4 tumor received a boost of external beam radiation of 9 Gy and 10 Gy directed to the primary tumor with another concurrent cycle of 5-FU and MMC for persistent disease. Ninety-five and 89% completed the two required cycles of 5-FU and MMC respectively.

### Tumor control and survival

Median follow-up time was 3 years (range 0.5-10 years). At 5 years, overall survival, disease-free survival (DFS) and colostomy-free survival rates for all 58 evaluable patients were 80%, 74%, and 94% respectively. Local control at five years according to T-Stage was T1-88%, T2-100%, T3-54% and T4-50%. The local and the regional control according to the node-status was N0-75% and 88% and N(1-3)-85% and 93% respectively. Among the eleven patients who had a local relapse, 7 had isolated local relapse and 4 had a synchronous local and regional relapse. Four out of 11 patients underwent salvage abdomino-perineal surgery without further relapse. Of the other 7 patients, 2 refused surgery and received palliative care, one was not a candidate for surgery, 3 were lost at follow-up and one has not yet been operated upon for his local relapse. Only two patients (3%) developed distant disease; a T1N0 patient developed liver metastasis and a T3N2 patient presented with bone metastasis.

### Prognostic factors

The variables tested at multivariate analysis were gender, HIV status, T3-T4 vs. T1-T2, N0 vs. N-positive, 0-1 cycle of 5-FU vs. ≥ 2 cycles of 5-FU and 0-1 cycle of MMC vs. ≥ 2 cycles of MMC. This analysis showed that stage T3-T4 was the only factor statistically associated with a worse local control and disease-free survival. This may be explained in part by the low number of patients per subgroups.

### Acute toxicity

Grade 3 and 4 skin toxicity, according to the RTOG scale, was reported in 81% and 2% of patients respectively. Ninety percent of women and 76% of men presented with grade 3 or 4 skin toxicity. Most of the acute gastrointestinal toxicity was grade 2 with only 2% and 4% reporting grade 3 and 4 toxicity respectively. There was no grade 3 or 4 genito-urinary toxicity. Eighty-six percent of patients reported no hematological toxicity while 7% had grade 3 or 4 toxicity.

### Quality of life (QOL)

At the time when the QOL questionnaires were sent out, 12 patients were deceased and 2 were alive with local recurrence. Fourteen patients were lost to follow-up (32%; 14/44 patients) due to the broad assessment time in which many patients had moved or were followed at another institution. Responses of both questionnaires were received from 30 patients and among them, there were no missing values. The median time between the last treatment of radiation therapy and the completion of the questionnaires was 51 (range 15-132) months. Ninety-five percent of patients answered the questionnaires at least 2 years after the end of the treatment.

The general EORTC QLQ-C30 functional and symptom scales and responses are shown in Table 2. Global functional QOL score was 70, with 100 being the best score. Functional aspects such as general physical and cognitive functions were excellent with scores above 80. Emotional, social and role functions seemed to be good after treatment with short split-course CRT. Gastro-intestinal symptoms including nausea/vomiting, constipation and/or appetite loss were reported to be very low, while diarrhea was frequently reported. Other symptoms commonly reported by the general population like fatigue, dyspnoea, pain, and financial problems were commonly reported. There were no changes over time in regard to the global functional QOL score, evaluated before or after 5 years from completion of the treatment.

**Table 2:** EORTC QLQ-C30

	CHUM N = 30 [Standard Deviation]	**GUH **[14] N = 41	**BCCA **[15]**Patients** N = 50	BCCA Volunteers N = 50	**Zurich **[16]**EBRT** N = 47	Zurich BT N = 34
**Functional scales**						
Global quality of life	70 [± 25]	71 [± 21]	66 [± 28]	78 [± 20]	86 [± 22]	72 [± 23]
Physical function	87 [± 14]	79.5 [± 22]	74 [± 29]	89 [± 14]	78 [± 27]	76 [± 31]
Role function	77 [± 26]	85 [± 21]	76 [± 33]	87 [± 25]	77 [± 32]	66 [± 37]
Emotional function	77 [± 26]	77 [± 25]	74 [± 28]	81 [± 16]	80 [± 24]	77 [± 25]
Cognitive function	85 [± 25]	76 [± 23]	75 [± 24]	82 [± 20]	75 [± 34]	78 [± 36]
Social function	74 [± 34]	82 [± 28]	73 [± 35]	90 [± 20]	77 [± 32]	70 [± 36]
						
**Symptoms scales**						
Fatigue	28 [± 29]	27 [± 22]	36 [± 30]	20 [± 21]	27 [± 9]	29 [± 11]
Pain	20 [± 25]	15 [± 21]	6 [± 17]	1 [± 14]	7 [± 2]	24 [± 15]
Nausea, vomiting	1 [± 4]	6 [± 15]	23 [± 15]	14 [± 22]	1 [± 0]	2 [± 0]
						
**Single items**						
Dyspnea	18 [± 26]	13 [± 22]	23 [± 33]	8 [± 16]	16 [± 7]	18 [± 7]
Sleep disturbance	26 [± 31]	23.5 [± 29]	29 [± 32]	22 [± 28]	29 [± 15]	33 [± 16]
Appetite loss	10 [± 23]	10 [± 19]	13 [± 22]	3 [± 15]	8 [± 3]	8 [± 3]
Diarrhea	28 [± 40]	28 [± 36]	27 [± 32]	5 [± 12]	24 [± 9]	29 [± 9]
Constipation	7 [± 19]	15 [± 21]	24 [± 32]	8 [± 16]		
Financial impact	20 [± 31]	15 [± 28]	23 [± 37]	8 [± 20	13 [± 6]	17 [± 10]

EORTC QLQ-C30 results of this study and of the literature. The questionnaire assesses cancer-specific QOL. For all the questions, a scale from 1 to 4 was used (1: not at all, 2: a little, 3: quite a bit, 4: very much). All scores were linearly transformed such that all scales range from 0 to 100. For the six functional items, the higher score represents a higher level of functioning and for the symptoms/single items, a higher score means a higher level of symptomatology/problems. Brackets indicate "standard deviation". N = number of patients.

The results from the disease-specific QLQ-CR29 (Table 3) revealed that 77% were satisfied with their body. This includes 60% to a great extent while 17% were a little dissatisfied. Twenty-three percent of patients were very much dissatisfied. The most common symptom was increased urinary frequency (40%), although only in 10% of patients to a maximum extent. Significant anal pain or fecal incontinence was rarely reported though 47% suffered some form of fecal incontinence. Only 17% complained of a disturbing involuntary loss of stool. Three patients with a stoma answered the questionnaire and they reported no problem in caring for their stoma. Fifty percent of patients maintained an interest in having sexual relations but 100% of male patients had difficulty maintaining an erection. Forty-four percent of men qualified the erectile dysfunction as severe (# 4 on the scale: very much). Among women, 65% had no interest at all in sexual relations, 21% a little, and only 14% had a moderate interest. For those women who maintained an interest in having sexual relations, 50% reported having pain or discomfort during intercourse. The majority of the patients did not suffer from non-satisfaction regarding their body or loss of masculinity or femininity in relation to their cancer or the treatment.

**Table 3:** QLQ-CR29

	1 Not at all	2 A little	3 Quite a bit	4 very much
Difficulty having or maintaining an erection	0%	44%	12%	44%
Fecal incontinence	53%	**30%**	7%	10%
Anal/perinal pain	60%	23%	10%	7%
Urinary frequency	33%	27%	30%	10%
Blood in stools	83%	17%	0%	0%
Feeling less feminine/masculine as a result of the disease or treatment	70%	7%	3%	20%
				
Dissatisfied with your body	60%	17%	0%	23%

EORTC QlQ-CR29 results of this study (n = 30 patients): The questionnaire assesses site specific (ano-rectal) QOL. For each questions, a scale from 1 to 4 was used (1: not at all, 2: a little, 3: quite a bit, 4: very much). n = number of patients.

## Discussion

Shortening the break inherent in split-course chemoradiation for anal cancer from 6 to 2 weeks has resulted in acceptable acute and chronic toxicities, though long-term QOL has yet to be evaluated for this regimen [14,15]. The EORTC phase II study 22953 demonstrated a severe long-term toxicity rate of 16% at 3 years from short split-course CRT. This included 4 patients suffering from ulceration and 1 patient from stenosis. Taking these results into consideration, we undertook a study designed to allow formal assessment of QOL in an unselected homogenous group of patients treated at our institution with split-course CRT. We report that such a short split-course CRT regimen is feasible with acceptable site specific (ano-rectal) long-term quality of life assessed with the QLQ-CR29 questionnaire. Increased urinary frequency and sexual dysfunction were the most frequent complaints. Five-year overall survival and colostomy-free survival for the whole group were very good approaching 80% and 94%, respectively. This is the first study using the QLQ-CR29 in addition to the QLQ-C30 questionnaire to evaluate QOL of patients with anal canal carcinoma treated with standard short split-course CRT.

There are some limitations to the current study. This study is a cross-sectional investigation of QoL with inherent limitations such as missing base-line QoL data and a bias due to different follow-up times. Results of QoL might differ when assessed after either a short or a long follow-up. The prevalence of missing data (32%) for the QLQ-C30 and the QLQ-CR29 questionnaires in this study appears to be high but is similar to other studies. The study of Allal et al. did not obtain the answers to the questionnaires of 11 out of 52 (21%) patients [14]. Jephcott et al. had a missing data rate of 45% (42/92 patients) [15]. The percentage of patients with T4 tumors in our series (22%) seems higher than in others (10-15%).

Three other studies evaluated QOL of anal cancer patients using the former EORTC QLQ-CR38 questionnaire after different treatment regimens (Allal et al., Jephcott et al., Oehler-Jänne et al.) [14-16]. These series used either 5.5 week split-course RT (11 patients) or CRT (30 patients) (Allal et al); 3.5 week split-course CRT (50 patients) compared to 50 healthy volunteers (Jephcott et al.); or 3 week split-course CRT (34 patients) compared with continuous CRT (47 patients) (Oehler-Jänne et al). In terms of general functioning and symptoms as evaluated by the QLQ-C30 questionnaire, our results are comparable with the results of the other 3 studies. Most important, overall QOL score and functional or symptom scores were good and not different from the scores of the healthy volunteer group reported by Jephcott et al. The site specific (ano-rectal) questionnaire QLQ-CR29 revealed that the majority of patients (77%) remained satisfied regarding their body in relation to their cancer or the treatment. Despite shortening the split, side-effects were infrequent with respect to significant involuntary loss of stool (17%) or anal pain (17%). These results are comparable with the observation by Allal et al, Jephcott et al and Oehler-Janne et al. where defecation problems were reported 18%, 20% and 21.4% respectively. Our subjective results are similar to the data from Vordermark et al. who evaluated continence by performing anorectal manometry in 16 patients with anal canal cancer treated with split course CRT [17]. Fifty-six percent were completely continent, 19% had liquid or solid soiling, and 6% were incontinent.

Our study showed that sexual dysfunction was very common. All men reported erectile dysfunction (100%) and only 50% maintained an interest in having sexual relations. For those women (35%) who maintained an interest in having sexual relations, 50% reported having pain or discomfort during intercourse. Accordingly, Allal et al. and Jephcott et al. as well showed a low score for sexual functioning, 13 and 24 (100 being the best score) respectively. Both groups used the EORTC QLQ-CR38 questionnaire. Only 35% (14/41 patients) in the study of Allal et al. reported some sexual activity. In our series, the median age of the male population was 52 which does not explain the high rate of erectile dysfunction. Erectile dysfunction (ED) is also found to be a common sequela after external beam radiotherapy and brachytherapy for prostate cancer [18]. It is controversial whether RT dose to sensitive structures like neurovascular bundles (NVBs), internal pudendal arteries (IPAs), accessory pudendal arteries, corpora cavernosa and the penile bulb is responsible for the high frequency of sexual dysfunction in the male population. So far, no correlation has been found between sexual dysfunction and RT dose to NVBs or IPAs and contradicting results regarding RT dose to corpora cavernosa and penile bulb. Our study as well as the studies mentioned above used an AP/PA technique whereby dose to the penile bulb may be assumed to be high. Sparing of the penile bulb could be achieved using intensity-modulated radiation therapy (IMRT) which is an investigational technique for the treatment of anal canal carcinoma and there is no long term QOL data available yet. On the other hand, sparing of the penile bulb to improve potency-preservation is not sufficiently supported by the current literature. In prostate cancer, use of IMRT compared favorably to previously reported series using conventional external beam radiation therapy techniques in preserving erectile function, although no correlation with RT dose was found [19]. In conclusion, shortening the radiation gap from 4-6 weeks to 12 days does not seem to result in worse QOL.

We were able to reproduce the good outcome of the EORTC phase II trial. Five-year overall survival and colostomy-free survival for the whole group in our series were 80% and 94%, as compared to 81% and 81% (at 3 years), respectively, in the EORTC study [6]. Results from randomized trials which used a 6 weeks split-course CRT regimen showed an overall survival ranging from 57% to 75% and a colostomy-free survival ranging from 71% to 72% [2-4].

Regarding side effects, severe gastrointestinal toxicity (4% vs. 12%) and hematological toxicity (7% vs. 2%) were comparable with the EORTC study. However, rate of acute skin toxicity was relatively high in our study with 81% (grade 3) and 2% (grade 4) as compared to 28% reported by Bosset et al. Total RT dose was not higher in our study (52 Gy vs. 59.4 Gy). Differences in the RT techniques might explain the increase in the severe dermatitis rate. We used elective RT to the groins whereas Bosset et al. irradiated the inguinal region only when the primary tumor was located less than 1 cm from the anal margin, or when the inguinal and/or the pelvic nodes were clinically positive [6]. A reduction in toxicities might be achieved using IMRT which has been shown in a recent study by Salama et al. where they reported grade 3 skin desquamation and grade 3 acute gastrointestinal toxicities in 38% and 15%, respectively [20].

Elective groin irradiation is controversial. While in North America, prophylactic inguinal irradiation is a routine practice and the RTOG protocols recommend 30.6 Gy in 17 fractions to this area, in Europe, it is not widely applied and the EORTC study by Bosset et al. did not apply it. The regional failure rate, either isolated or associated with a local or a distant relapse, was 12%. It has to be pointed out that in our study, prophylactic dose to microscopic disease including groins was rather small with a median dose of 24 Gy (range 20-30 Gy). Inguinal relapse was observed in 3 of 58 patients (5%) without groin involvement at the time of initial presentation (1 patient T1N0 and 2 patients T4N0). Similar inguinal failure rates have been reported after CRT including elective groin irradiation in a series of 276 patients at the Institut Curie de Paris and at other institutions [21-24].

The prospective non-randomized study from Cummings et al. reported the results of different radiation therapy and chemotherapy protocols [25]. Fifty-three out of 192 patients received split course CRT with 5-FU and MMC (2 × 25 Gy: 14 patients, 2 × 24 Gy: 33 and 3 × 20 Gy: 6). In this subgroup, the regional recurrence was 9%. The EORTC trial gave 45 Gy to microscopic disease combined with 5-FU and MMC followed by a boost of 15 Gy or 20 Gy to macroscopic disease for complete and partial responders respectively, after a break of six weeks [2]. The loco-regional relapses were 32% and the majority had an isolated local recurrence. Prophylactic radiation therapy of 24 Gy combined with 5-FU and MMC seems adequate to control microscopic disease. However, after CRT without elective RT to the groins, metachronous inguinal metastases have been reported in only 19 out of 243 patients (7.8%) in a series of 270 from Gerard et al. [26]. The EORTC study 22953 reported no inguinal failure. The low rate of inguinal relapses found in this series could also be explained by low propensity of this disease to cause inguinal metastases even without prophylactic irradiation.

## Conclusion

In conclusion, short split-course CRT for anal canal carcinoma seems to be associated with good long-term global QOL, though increased micturition and sexual dysfunction remained important problems. Local control, colostomy-free and overall survival were excellent but acute skin toxicity noticeably high. Implementation of modern RT techniques such as IMRT and reduction of RT dose to the groins might have the potential to improve acute toxicity and late morbidity.

## Competing interests

The authors declare that they have no competing interests.

## Authors' contributions

S.P. carried out conception and design, collection and assembly of data, data analysis, manuscript writing, C.O. carried out data analysis and interpretation, manuscript writing, S.L. carried out manuscript writing, data interpretation, B.F. carried out data interpretation, manuscript writing, M.J. manuscript writing, D.D. carried out conception and design, financial support, data analysis and interpretation, manuscript writing. All authors read and approved the final manuscript.
